# Awareness and treatment of chronic hepatitis B in Malaysia-findings from a community-based screening campaign

**DOI:** 10.1186/s12876-022-02458-9

**Published:** 2022-08-06

**Authors:** Zhuo-zhi Lim, Jau shya Teo, Ah-Choon Tan, Teck Onn Lim

**Affiliations:** Hepatitis Free Pahang Malaysia, D7-3-1, Pusat Perdagangan Dana 1, Jalan PJU 1A/46, 47301 Petaling Jaya, Selangor Malaysia

**Keywords:** Cascade of care, Awareness, Treatment, Hepatitis B, Malaysia

## Abstract

**Introduction:**

In 2016 the World Health Organization (WHO) had adopted a global strategy to eliminate Hepatitis B (HBV) by 2030 through five core interventions. One of which is the “cascade of care”, the continuum of services that persons with chronic Hepatitis B Virus (HBV) should receive as they progress from screening to diagnosis to treatment to chronic care. We determined the prevalence of the awareness and treatment of chronic HBV in Malaysia based on a large sample data from a screening campaign.

**Methods:**

A total of 10,436 subjects participated in the HBV screening campaign organized by the Hepatitis Free Pahang Malaysia (HFP). Between in 2018 and 2019, HFP organized a total of 109 health fairs in partnership with local non-governmental organizations (NGO) to conduct HBV screening mostly in small towns and villages largely in the state of Pahang. All screen-positive subjects were recalled to undergo laboratory-based HBsAg and HBV DNA tests. Patients with confirmed chronic HBV were referred to local health services, while continued being monitored by HFP.

**Results:**

We estimated 13.1% of Malaysian adults aged 20 or older with chronic HBV were aware of their HBV status, and of those only 0.7% had received prior anti-viral treatment, but among those with baseline HBV DNA level > 20,000 IU/ml, 15.6% were subsequently treated. Tenofovir disoproxil fumarate was the only medicine used on all treated patients.

**Conclusion:**

Few Malaysian adults with HBV were aware of their infection and even less received anti-viral therapy. Concerted public health efforts are urgently needed to improve HBV screening and care cascade in order to meet WHO’s targets for HBV elimination.

## Introduction

Chronic hepatitis B virus (HBV) infection is endemic in the Asia pacific region [1] including in Malaysia. A recent survey [2] estimated a prevalence of 1.2% amongst the multi-ethnic Malaysian population; the disease is particularly common among Chinese with a prevalence of 2.2%. In Malaysia, cirrhosis ranks ninth among the top ten major disease burden [3], while hepatocellular carcinoma is not only common (5th commonest cancer), it is also the most deadly cancer in Malaysia [4].

In 2016 the World Health Organization (WHO) adopted a global strategy to eliminate HBV and HCV by 2030 [5]. The “cascade of care” refers to the continuum of services that persons with chronic HBV should receive as they progress from screening to diagnosis to treatment to chronic care. It is one of five core interventions to eliminate HBV. However most people with chronic HBV remain undiagnosed and untreated globally. The WHO estimated only 9% of people were aware of their chronic HBV, and of those only 8% received anti-viral therapy [5].

In this study, we provide estimates of the prevalence of HBV awareness and treatment in Malaysia based on a large sample data derived from community-based screening campaigns in 2018 and 2019 organized by Hepatitis Free Pahang (HFP) [6]. HFP is a Malaysia-based Non-Governmental Organization (NGO) established in 2017 in response to the continuing under-development of screening and treatment services for chronic HBV in Malaysia. With the availability of low cost generic oral Tenofovir, it is becoming urgent to scale up chronic HBV screening and treatment services in Malaysia. The mission of HFP is to mobilize local community to raise awareness about HBV, to provide free public screening services and to improve access to treatment for chronic HBV.

## Methods

Study sample for this study are people who attended the screening campaigns organized by the Hepatitis Free Pahang (HFP). The Ministry of Health’s (MOH) Medical and Research Ethics Committee approved the study and all subjects gave informed consent.

Between in 2018 and 2019, HFP organized a total of 109 health fairs to conduct screening mostly in small towns and villages and largely in the state of Pahang. All attendees at these health fairs registered online to participate in the screening tests. The online data system helped to support the conduct of the screening and administer questionnaire, to manage screen-positive subjects for subsequent testing and counselling, to facilitate reporting of results through short messaging service and to help capture the data for this study. Questionnaire used in this survey is available from the authors on request.

We used a low-cost point-of-care HBsAg test (POCT, AllTest Biotech) to screen for HBV. The POCT have previously been validated [7]. All screen-positive subjects were subsequently recalled to undergo confirmatory testing, which were lab based serology and HBV DNA tests. A trained nurse would counsel patients confirmed to have chronic HBV on infection transmission, risk of liver disease progression, need for monitoring and treatment. All HBsAg + individuals were referred to the local health service for further care if they were not already receiving care. All individuals were also offered free monitoring of their HBV DNA levels. Through this, the trained nurses continue to follow-up on patients’ treatment and outcome.

We define chronic HBV patient as those who were screened and subsequently re-tested positive on lab-based HBsAg test. Awareness of HBV is defined as answering “yes” to the nurse-administered question: “Have you ever been tested or told by a doctor that you have hepatitis B?”, while Prior treatment of chronic HBV is defined as answering “yes” to the question: “Were you or are you currently on anti-viral treatment prescribed by a doctor?”, and corroborated by enquiry on the name of prescribing provider, the name of the medicine(s) and on previous HBV DNA test results.

Participants in the screening campaign composed of a convenience sample which is not representative of the general population (older subjects, Chinese and Pahang residents were over-represented compare to the population). To estimate the population prevalence, post stratification [8] was used to adjust the sample totals to known population totals for age, gender and ethnicity based on the Population And Housing Census of Malaysia in 2010.

## Results

A total of 10,436 subjects participated in the HBV screening campaigns in 2018 and 2019, of whom 200 were identified to have chronic HBV, their mean age was 52 years, with equal number of males and females.

Of the 200 subjects with chronic HBV, only 2 (1%) had prior anti-viral treatment.

Post-screening, only 108 (54%) of these 200 screen positive subjects had returned to have their HBV DNA measured. Their median HBV DNA was 10,804 IU/ml, 37% had levels below 2000 IU/ml while 41% had levels above 20,000 IU/ml. Only 115 (58%) individuals had subsequently sought care and were on follow-up.

The prevalence of awareness among Malaysian adults age 20 or older with chronic HBV was 13.1%. The prevalence of prior anti-viral treatment in the screen positive chronic HBV population was only 0.7%. Of those with baseline HBV DNA > 20,000 IU/ml, 15.6% had subsequently received anti-viral treatment on follow-up. All were treated with Tenofovir disoproxil fumarate. There is no apparent trend in the prevalence of awareness or treatment by age and sex (Table 1).

**Table 1:** Prevalence of Awareness and Anti-viral treatment among subjects with chronic HBV in Malaysia 2018–2019

	Awareness		Treatment	
	Number with chronic HBV = 200		Number with chronic HBV and HBV DNA > 20,000 IU/ml = 43	
	Number who were aware of their chronic HBV = 33		Number who subsequently received treatment on follow-up = 6	
	Prevalence	95% CI	Prevalence	95% CI
All	13.1	(7.7, 18.5)	15.6	(0, 31.2)
*By Age*				
20–39 years	7.5	(0, 15.8)	17.9	(0, 41.1)
40–54 years	23.9	(14.7, 33.1)	0	–
> = 55 years	13.6	(5.7, 21.5	28.8	(3.9, 53.7)
*By Gender*				
Male	14.1	(6.3, 21.9	5.6	(0, 12.4)
Female	11.9	(4.6, 19.3	25.8	(0, 54.8)

## Discussion

This study shows that few Malaysian adults were aware (13.1%) of their infection and even less had prior anti-viral therapy (0.7%), though among those with HBV DNA > 20,000 IU/ml, 15.6% had subsequently received treatment. This is much lower than in the US, where the NHANES survey had found awareness and prior treatment rates of 32% and 28% respectively [9]. A study from China based on insurance database found that treatment rates has increased from 3.9% in 2010 to 30.9% in 2018 [10].

Clearly the “cascade of care” for chronic HBV in Malaysia is broken. There remains a huge gap between our health system’s performance and the WHO targets of 90% diagnosis and 80% treatment rates required to eliminate HBV [5]. Concerted public health efforts are urgently needed. We provide some suggestions to improve HBV care in Malaysia:

- Access to HBV DNA testing to guide treatment. Only 2 screen- positive subjects had prior HBV DNA levels measured, a test which is still prohibitively costly to most patients in Malaysia. We need low-cost test, alongside a low-cost HBV treatment like generic Tenofovir which drove the higher prospective treatment rate observed in this study.
- Adequately resourced and prepared local health services to deliver chronic HBV care. In Malaysia, patients with chronic HBV have hitherto been managed by Hepatologists and Gastroenterologists, the supply of these specialists are very limited. For example, Pahang, where this screening campaign was largely conducted, has a population of 1.6 million, but only one Gastroenterologist in the public hospital and 2 in private, all were located faraway in the capital city Kuantan. This represents a bottle-neck in the cascade of care which would lead to delays in HBV follow up. In line with current WHO guideline [11], we will need to mobilize the local primary care workforce where the campaign is conducted to deliver the medical care locally for patients with uncomplicated chronic HBV.
- Non-adherence. In this study, only 54% of screen-positive subjects who were offered free HBV DNA had come forward to be tested. And only 58% of subjects with chronic HBV had remained on follow-up with a healthcare provider despite repeated calls and reminders. Likewise, there is likely non-adherence on the part of healthcare providers [12] though this study was not designed to address this issue. Non-adherence is clearly a significant cause of failure in the linkage to care. There is room for improvement in the design and responsiveness of our health services to the needs of patients with chronic disease like HBV [13]. Similarly, we need to begin to address patients’ ignorance about HBV and the stigmatization associated with HBV, and other social and/or cultural barriers to patients seeking care [14–16], though again this study was not designed to address these issues.

Our study has several limitations. First, the study subjects were not a probability sample and are not representative of the population. They were older than the general population, and Chinese and Pahang residents were over-represented as a result of the conduct of the campaign through partnership with local NGOs, most of which were faith or ethnic based organizations in Pahang. Post-stratification was required to adjust the sampling weight to reflect the age, sex and ethnicity distribution of Malaysia. Second, subjects known to have hepatitis may be more or less willing to participate in screening. This source of bias applies to probability sample too; such subject may be more or less willing to consent to be tested in a sampling survey. The risk of this bias however is lessened in our study by pooling the data from numerous (109) screening events or venues conducted in numerous rural and urban locations spread over a wide geographical region. Third, the determination of awareness and treatment were based on self-report by patient, as is usual in all sample surveys. In our study however, instead of just accepting a Yes/No response to survey questions on awareness and treatment at face value, we had required corroborative information on the circumstances and details on names of provider/treatment and test results before accepting a”Yes” response as indicating awareness or treatment.

## Data Availability

The datasets used and/or analysed during the current study are available from the corresponding author on reasonable request.

## Abbreviations

CI: Confidence interval
HBV: Hepatitis B virus
HCV: Hepatitis C virus
HFP: Hepatitis free Pahang
MOH: Ministry of health Malaysia
NGO: Non-governmental organizations
NHANES: National health nutrition examination survey, US
POCT: Point of care tests
WHO: World Health Organization
